# Imaging Techniques in Acute Coronary Syndromes: A Review

**DOI:** 10.5402/2011/359127

**Published:** 2011-11-17

**Authors:** Stanley K. Zimmerman, James L. Vacek

**Affiliations:** Division of Cardiovascular Diseases, University of Kansas Hospital and Medical Center, 3901 Rainbow Boulevard, 1001 Eaton Mail Stop 3006, Kansas City, KS 66160, USA

## Abstract

Coronary heart disease (CHD) remains the leading cause of death in the United States. National review of Emergency Department (ED) visits from 2007 to 2008 reveals that 9% are for chest pain. Of these patients, 13% had acute coronary syndromes (ACSs) (Antman et al., 2004). Plaque rupture with thrombus formation is the most frequent cause of ACS, and identifying patients prior to this event remains important for any clinician caring for these patients. There has been an increasing amount of research and technological advancement in improving the diagnosis of patients presenting with ACS. Low-to-intermediate risk patients are the subgroup that has a delay in definitive treatment for ACS, and a push for methods to more easily and accurately identify the patients within this group that would benefit from an early invasive strategy has arisen. Multiple imaging modalities have been studied regarding the ability to detect ischemia or wall motion abnormalities (WMAs), and an understanding of some of the currently available noninvasive and invasive imaging techniques is important for any clinician caring for ACS patients.

## 1. Introduction

CHD continues to remain the leading cause of death in the United States (USA), and the prevalence has increased worldwide as diets continue to “westernize”. Of the nearly 5.5 million visits annually for chest pain, <10% of patients present with ST segment elevation [1], and of the remaining patients only *∼*13% have ACS [2]. There is significant morbidity and mortality associated with ACS and a significant cost in excluding it. National figures estimate *∼*10 billion dollars are spent annually in excluding ACS in the USA. As economic markets and turmoil continue to affect the healthcare landscape, there is increased scrutiny on methods to control cost while improving diagnosis. Physicians and scientists worldwide have thus sought ways to improve the accuracy and speed of diagnosis by studying methods that detect coronary artery disease (CAD) either by identifying decreased perfusion or the consequences of decreased perfusion. Methods aimed at detecting ischemia or regional wall motion abnormalities (RWMAs). Patients with ACS are increasingly being managed with invasive strategies, but methods to identify patients that would benefit from this strategy earlier in their presentation are vital to improve care. Imaging tools that have been evaluated include noninvasive measures like contrast echocardiography, multidetector computed tomography (MDCT), single photon emission tomography (SPECT), nuclear perfusion imaging, positron emission tomography (PET), cardiac magnetic resonance imaging (CMR), and even invasive techniques like intravascular ultrasound (IVUS) and optical coherence tomography (OCT). The most effective tool would be very specific and sensitive, rapid, cost-effective, noninvasive, and able to be performed bedside. None of the aforementioned tools fulfill all of these, but they all have significant roles and provide important diagnostic information that aides the clinician in the diagnosis of ACS. A review of these imaging modalities and their strengths and weaknesses is important information for any clinician managing patients with ACS.

Analysis by Fox et al. of the global registry of acute coronary events (GRACEs) revealed that between 1999 and 2006 improvements in managements of patients with ACS were associated with a decrease in the rates of heart failure and mortality [3]. Rates of cerebrovascular accidents and myocardial infarction were reduced. During this time, rates of percutaneous coronary interventions (PCIs) increased by *∼*18%. The 2011 update in patients with ACS supports an early invasive strategy for those with refractory angina or continued instability and in those elevated risk as a class I recommendation. Meta-analyses of early invasive strategies have revealed that routine invasive strategies had an 18% relative reduction in death or myocardial infarction (MI), and a significant reduction in MI alone [4]. Experience and evidence shows that patients presenting with ST segment elevation myocardial infarction (STEMI), high-risk unstable angina (UA), and non-ST segment elevation myocardial infarction (NSTEMI) benefit from an early invasive strategy. Angiography remains the gold standard for these patients. The question remains regarding low- and intermediate-risk patients and the most effective way of establishing whether CHD is present or absent. No currently available tool is both 100% sensitive and specific for ACS. A summary of the imaging modalities that currently available as well as strengths and limitations are listed in Table 1 and will be discussed further. The electrocardiogram (EKG) is still one of the most important methods a clinician has in making this early distinction, but the EKG does little to establish a diagnosis unless positive in the acute setting. 

## 2. Echocardiography

Echocardiography can rapidly assess for RWMA, is highly portable, and relatively inexpensive compared with other noninvasive modalities. Echocardiography relies on detecting wall motion changes, which occur when myocardial blood flow falls below resting levels. Often this occurs when coronary obstruction exceeds 85–90% of the luminal area [5]. Myocardial blood flow affects myocardial oxygen consumption. When there is a supply-demand mismatch, myocardial contractility is affected and leads to the development of hypokinesis [6]. Echocardiography is a class I indication to evaluate RWMA in patients presenting with chest pain but with low-to-intermediate risk. Earlier discussions have revealed that echocardiography had a high sensitivity (92-93%) in detecting AMI and cardiac ischemia (88%) in patients presenting to the ED with chest pain [7]. The specificity was only 53–57% in this same group. The addition of contrast echocardiography has been employed to improve these parameters. Myocardial contrast echocardiography (MCE) has been used to evaluate RWMA and to assess microvascular perfusion. A decrease in myocardial blood flow (MBF) results in a reduction in myocardial blood volume, and with MCE a perfusion defect can be visualized. Evaluation of MCE in a multicenter study when compared with single photon emitted computed tomography found that the two were similar in their ability to identify and diagnose those with AMI [8]. Lønnebakken et al. [9] used MCE to prioritize patients with NSTEMI and angiographically severe CAD. Their study used a wall motion score compromising the 17 segments left ventricular model and myocardial perfusion followed by CAD assessment invasively measured by quantitative coronary analysis (QCA). Results from this study found that patients with ≥6 hypoperfused left ventricular segments had a 7-fold higher risk of severe CAD. Their findings were not specific for the area of stenosis unless it involved the proximal left anterior descending (LAD) coronary artery. Limitations in echocardiography are often the result of limited or poor acoustic windows, its poor specificity, operator-dependent acquisition, and is one of the most subjective imaging modalities.

## 3. Computed Tomography

MDCT has been studied extensively to determine its role in the noninvasive assessment of ACS. MDCT has the ability to identify plaque area and the degree of stenosis. The sensitivity of MDCT to detect CAD has been reported to be 73–100% with a specificity of 91–97% [10–12]. MDCT has good correlation with IVUS [13] and coronary angiography [14]. A study by Hoffmann et al. [15] used MDCT to compare lesion characteristics in culprit lesions from patients with ACS, stable lesions in patients with ACS, and stable lesions in patients with stable angina. Lesions that were detected by MDCT correlated well to coronary angiography thus possibly serving as a method to further risk stratify patients with ACS. All lesions that had impaired image quality were not included in the analysis. MDCT studies have also sought to define the high-risk characteristics in lesions that may make patients more susceptible to ACS. Detection of low attenuation of <30 Hounsfield units and positive remodeling has proven very accurate in predicting future ACS [16]. Recent work [17] has shown that MDCT can predict thin cap fibroatheromas (TCFA) and vulnerable plaque by identifying low attenuation, a large remodeling index, and a signet ring-like appearance. These predictors correlated well with OCT. Motoyama et al. [16] studied coronary artery lesions in patients with NSTEMI, STEMI, or unstable angina presenting >24 hours after symptoms onset whom were symptom-free and hemodynamically stable. Culprit lesions in patients with ACS are characterized by large plaque volume, necrotic cores and local inflammation. MDCT studies with IVUS and OCT have also confirmed that high attenuation around the coronary artery plaque was more susceptible to rupture [18]. MDCT is limited by its inability to image lesions with heavy calcium burden, necessitates the use of intravenous contrast, and requires radiation exposure. The greatest asset of MDCT may be the negative predictive value for excluding significant CAD in those at low-to-intermediate risk presenting with chest pain.

## 4. Cardiac Magnetic Resonance Imaging

The role of CMR has clear roles in the congenital heart disease, chronic CAD, myocardial and pericardial diseases, and imaging of the great vessels. CMR has a sensitivity of 84% and a specificity of 85%, which is greater than EKG or troponin and more specific than an abnormal troponin [19]. There are multiple methods that CMR employs to establish a diagnosis of ACS. CMR cine imaging can assess global and regional left ventricular function, and its accurate and reproducible ventricular volumes and functions make it more accurate than other noninvasive methods [20]. First pass myocardial perfusion utilizes a contrast agent coadministered with a vasodilator-like adenosine to delineate under perfused areas highlighting subendocardial ischemia. The MR-IMPACT study evaluated 234 patients with CMR and SPECT, and CMR was better at detecting coronary artery stenosis than SPECT [21]. CMR has been used to evaluate microvascular obstruction (MVO). Imaging within the first few minutes of contrast administration can detect MVO by revealing decreased contrast delivery to the infarcted area and decreased signal intensity on T1-weighted imaging. Myocardium that is acutely or chronically infarcted that does not have MVO will retain contrast and have bright signal intensity on T1-weighted images. Viability can be assessed utilizing CMR via late gadolinium enhancement. Ten to twenty minutes after contrast administration, delayed imaging can highlight the extent of the scar and potential for functional recovery. CMR can also detect myocardial edema often present in acute injury with T2-weighted images, and coronary CMR angiography can detect proximal coronary artery stenosis. The evaluation of CMR as an imaging modality for the stratification of patients presenting to the ED with chest pain has been extensively evaluated, and it can identify ACS more predictably than EKG, troponin, and TIMI score [19]. Plein et al. [22] studied NSTEMI patients 2–5 days after presentation and concluded that CMR reliably predicted coronary stenosis necessitating revascularization confirmed by angiography. CMR also can fill a vital role in differentiating ACS from other myocardial diseases such as myocarditis and can detect complications from ACS such as left ventricular thrombus, ventricular septal defects, and aneurysms [23]. Despite advances in CMR techniques several limitations persist. Acquisition times are lengthy, unstable patients and patients without new generation pacemakers or with metallic fragments are not candidates, and it is relatively expensive, and is not portable. CMR with its limitations still remains a viable option in the diagnosis and stratification of patients presenting with chest pain.

## 5. Positron Emission Tomography

PET with 18F-fluorodeoxyglucose (FDG) is able to identify functional metabolic activity by imaging glucose utilization. FDG once injected is taken up by cells that utilize glucose for metabolism, and the more metabolic activity of the tissue the greater the amount of FDG, taken up. As FDG decays gamma rays are emitted and the position of origin is imaged by PET imaging. The majority of FDG PET for cardiac applications is assessing viability, but interest in its use for ACS has arisen due to its high sensitivity for molecular targets. Imaging with PET in ACS relies on the ability to detect acute inflammation. Atherosclerotic coronary plaques are characterized by macrophage accumulation. FDG uptake is increased in these areas as macrophages often take up more glucose than the surrounding tissues [24, 25]. PET has limited spatial resolution of 3–5 mm making reproducible measurements in the right coronary artery (RCA) and mid to distal vessels somewhat problematic. PET is further limited as it uses ionizing radiation, requires coregistration with CT or MRI for localization, and is hindered by cardiac and respiratory motion [26, 27]. Studies employing a diet rich in free fatty acids prior to imaging leads to a decrease in myocardial uptake of tracer without affecting other tissue improving coronary artery imaging [28]. PET, due to its ability to detect increased metabolic activity, may help identify vulnerable high risk plaque that is not obstructive to blood flow due to positive remodeling, but still prone to rupture and subsequent ACS.

## 6. Single Photon Emission Computed Tomography

Myocardial perfusion imaging has long been used for the detection of ischemia and even viability. Resting and stress myocardial perfusion imaging in patients with low-to-intermediate risk for CAD will identify active inducible ischemia; however, in patients with recent angina symptoms SPECT may not be able to identify recent and old infarcts limiting its specificity. SPECT is more sensitive than exercise treadmill testing alone for detecting coronary artery stenosis of >50% with a sensitivity of 87% and a specificity of 73%, and with vasodilator stress the sensitivity is 89% and specificity is 75% [29]. There is much less operator dependency with SPECT imaging, and simultaneous assessment of regional perfusion and function can be obtained. A study performed in patients with chest pain randomization to resting SPECT did not alter or affect treatment decisions in patients with an eventual diagnosis of AMI or UA. SPECT did reduce the rate of admissions in those without ACS [30]. Limitations with SPECT are that it has imaging protocols up to 4 hours for stress rest comparison, necessitates radiation exposure, has a lower spatial resolution than echocardiography, can underestimate three vessel coronary artery disease due to balanced ischemia, and can produce many attenuation artifacts.

## 7. Invasive Imaging Techniques: IVUS and OCT

Several invasive methods exist to detect and characterize vulnerable plaque in patients with CHD and IVUS, and OCT will be discussed further here. Our understanding of plaque progression and vulnerable plaque characteristics has been greatly enhanced with IVUS/IVUS virtual histology (VH) and OCT. In the PROSPECT study, patients presenting with ACS underwent coronary angiography and IVUS after PCI. During the 3 year followup, there was a 20% risk of major adverse cardiovascular events (MACEs) including 13% in culprit lesions and 12% in nonculprit lesions [31]. The greatest predictors of this risk were a large plaque burden of ≥70%, minimal luminal area (MLA) ≤4 mm by IVUS with large necrotic core or TCFA. The highest risk was in patients with TCFA, increased plaque burden, and decreased MLA. Patients with long plaques *∼*12 mm had increased risk as well. IVUS allows in vivo wall visualization and the differentiation of morphological characteristics between culprit and nonculprit plaques [32]. In a study with serial IVUS imaging over 12 months *∼*75% of TCFA heal during followup or evolve to less vulnerable plaques. TCFA may develop from pathological intimal thickening and thick cap fibroatheromas (ThCFA), and disease progression is more likely with increased plaque volume [33]. Limits in IVUS utilization are that it is invasive and thus not ideal for those not undergoing angiography. IVUS has the inability to detect TCFA (<65 *μ*m) due to its limited spatial resolution of >100 *μ*m, and an inability to accurately distinguish plaque components [34]. OCT, on the other hand, has high resolution of 10 to 20 *μ*m, *∼*10 times that of IVUS [35]. In ACS, OCT has identified culprit lesions in STEMI patients when compared to NSTEMI patients who have more plaque rupture, TCFA, and red thrombus. Unstable plaques are defined by TCFA. A cap of <65 *μ*m has been observed in 95% of ruptured plaques [36]. OCT is able to identify rupture-prone plaques by clearly visualizing large lipid cores, TCFA, and assess inflammation [37]. OCT is limited as it is invasive, has limited depth penetration making it poor at delineating lipid content, and is time consuming in that each coronary artery must be imaged separately. Although IVUS and OCT require an invasive approach to detect high-risk plaque characteristics they have been vital in our understanding of these high-risk indicators and have enabled further characteristics to be defined by noninvasive measures.

## 8. Summary

The accurate evaluation, diagnosis, and risk stratification of patients presenting to the clinical setting with chest pain remains one of the most important clinical processes. ACS affects millions of people worldwide with costs of evaluating these patients in the billions. Research and clinical studies continue to seek for methods that are cost effective, portable, and highly reliable at establishing a diagnosis more efficiently so that patients can receive the fastest available treatment (see Table 1). Several noninvasive measures are available to the clinician with strengths and weaknesses for each. As processes in the ED and other clinical settings become more streamlined, a team-dedicated approach aimed at providing best available care is the most important. Protocols regarding ACS medications, timeliness of EKG's, and stratification schemes regarding patients risk profile are the most important tools at hand to provide this care. In the subgroup of patients that are then determined to be low-to-intermediate risk noninvasive methods should be utilized in the timeliest manner to further stratify these patients to an early invasive or more conservative strategy. Clinicians should seek to use the methods at their facility that are the most accurate, cost effective, and provide the greatest diagnostic information to provide their patients with the best available care.

## Figures and Tables

**Table 1:** ACS imaging modalities.

	Cost	Invasiveness	Portability	Timeliness	Main limitations
Echocardiography	+	+	+++	+++	Poor acoustic windows, low specificity, operator-dependent acquisition, subjective
Computed tomography	++	+	+	+++	Inability to image lesions with heavy calcium burden, IV contrast, radiation exposure
Cardiac MRI	+++	+	+	+	Acquisition time, unstable patients, metallic fragments
Positron emission tomography	+++	+	+	+	Limited spatial resolution, ionizing radiation, requires coregistration with CT or MRI, hindered by cardiac and respiratory motion
Single photon emission tomography	++	+	+	+	longer imaging protocols for stress rest comparison, radiation exposure, lower spatial resolution than echocardiography underestimate three vessel CAD due to balanced ischemia, attenuation artifacts
IVUS/OCT	+++	+++	+	+	Time consuming each coronary artery must be imaged separately, limited spatial resolution of >100 *μ*m with IVUS, limited depth penetration with OCT

Key: + least, ++ moderate, +++ most.

## References

[1] Antman EM, Anbe DT, Armstrong PW (2004). ACC/AHA guidelines for the management of patients with ST-elevation myocardial infarction: a report of the American College of Cardiology/American Heart Association Task Force on Practice Guidelines. *Journal of the American College of Cardiology*.

[2] Bhuiya FA, Pitts SR, McCaig LF (2010). Emergency department visits for chest pain and abdominal pain: United States, 1999–2008. *National Center for Health and Statistics Data Brief*.

[3] Fox KAA, Steg PG, Eagle KA (2007). Decline in rates of death and heart failure in acute coronary syndromes, 1999–2006. *Journal of the American Medical Association*.

[4] Mehta SR, Cannon CP, Fox KAA (2005). Routine vs selective invasive strategies in patients with acute coronary syndromes: a collaborative meta-analysis of randomized trials. *Journal of the American Medical Association*.

[5] Gould KL, Lipscomb K (1974). Effects of coronary stenoses on coronary flow reserve and resistance. *American Journal of Cardiology*.

[6] Leong-Poi H, Coggins MP, Sklenar J, Jayaweera AR, Wang XQ, Kaul S (2005). Role of collateral blood flow in the apparent disparity between the extent of abnormal wall thickening and perfusion defect size during acute myocardial infarction and demand ischemia. *Journal of the American College of Cardiology*.

[7] Sabia P, Afrookteh A, Touchstone DA, Keller MW, Esquivel L, Kaul S (1991). Value of regional wall motion abnormality in the emergency room diagnosis of acute myocardial infarction. A prospective study using two-dimensional echocardiography. *Circulation*.

[8] Kaul S, Senior R, Firschke C (2004). Incremental value of cardiac imaging in patients presenting to the emergency department with chest pain and without ST-segment elevation: a multicenter study. *American Heart Journal*.

[9] Lønnebakken MT, Staal EM, Nordrehaug JE, Gerdts E (2011). Usefulness of contrast echocardiography for predicting the severity of angiographic coronary disease in nonST-elevation myocardial infarction. *American Journal of Cardiology*.

[10] Abdulla J, Abildstrom SZ, Gotzsche O, Christensen E, Kober L, Torp-Pedersen C (2007). 64-Multislice detector computed tomography coronary angiography as potential alternative to conventional coronary angiography: a systematic review and meta-analysis. *European Heart Journal*.

[11] Bluemke DA, Achenbach S, Budoff M (2008). Noninvasive coronary artery imaging: magnetic resonance angiography and multidetector computed tomography angiography: a scientific statement from the American Heart Association committee on cardiovascular imaging and intervention of the council on cardiovascular radiology and intervention, and the councils on clinical cardiology and cardiovascular disease in the young. *Circulation*.

[12] Vanhoenacker PK (2007). Diagnostic performance of multi-detector CT angiography for assessment of coronary artery disease: meta-analysis. *European Heart Journal *.

[13] Moselewski F, Ropers D, Pohle K (2004). Comparison of measurement of cross-sectional coronary atherosclerotic plaque and vessel areas by 16-slice multidetector computed tomography versus intravascular ultrasound. *American Journal of Cardiology*.

[14] Cury RC, Pomerantsev EV, Ferencik M (2005). Comparison of the degree of coronary stenoses by multidetector computed tomography versus by quantitative coronary angiography. *American Journal of Cardiology*.

[15] Hoffmann U, Moselewski F, Nieman K (2006). Noninvasive assessment of plaque morphology and composition in culprit and stable lesions in acute coronary syndrome and stable lesions in stable angina by multidetector computed tomography. *Journal of the American College of Cardiology*.

[16] Motoyama S, Kondo T, Sarai M (2007). Multislice computed tomographic characteristics of coronary lesions in acute coronary syndromes. *Journal of the American College of Cardiology*.

[17] Ito T, Terashima M, Kaneda H (2011). Comparison of in vivo assessment of vulnerable plaque by 64-slice multislice computed tomography versus optical coherence tomography. *American Journal of Cardiology*.

[18] Kashiwagi M, Tanaka A, Kitabata H (2009). Feasibility of noninvasive assessment of thin-cap fibroatheroma by multidetector computed tomography. *Journal of the American College of Cardiology*.

[19] Kwong RY, Schussheim AE, Rekhraj S (2003). Detecting acute coronary syndrome in the emergency department with cardiac magnetic resonance imaging. *Circulation*.

[20] Bellenger NG, Burgess MI, Ray SG (2000). Comparison of left ventricular ejection fraction and volumes in heart failure by echocardiography, radionuclide ventriculography and cardiovascular magnetic resonance. Are they interchangeable?. *European Heart Journal*.

[21] Schwitter J, Wacker CM, Van Rossum AC (2008). MR-IMPACT: comparison of perfusion-cardiac magnetic resonance with single-photon emission computed tomography for the detection of coronary artery disease in a multicentre, multivendor, randomized trial. *European Heart Journal*.

[22] Plein S, Greenwood JP, Ridgway JP, Cranny G, Ball SG, Sivananthan MU (2004). Assessment of non-ST-segment elevation acute coronary syndromes with cardiac magnetic resonance imaging. *Journal of the American College of Cardiology*.

[23] Lockie T, Nagel E, Redwood S, Plein S (2009). Use of cardiovascular magnetic resonance imaging in acute coronary syndromes. *Circulation*.

[24] Björnheden T, Levin M, Evaldsson M, Wiklund O (1999). Evidence of hypoxic areas within the arterial wall in vivo. *Arteriosclerosis, Thrombosis, and Vascular Biology*.

[25] Mayr M, Sidibe A, Zampetaki A (2008). The paradox of hypoxic and oxidative stress in atherosclerosis. *Journal of the American College of Cardiology*.

[26] Rogers IS, Tawakol A (2011). Imaging of coronary inflammation with FDG-PET: feasibility and clinical hurdles. *Current Cardiology Reports*.

[27] Fox JJ, Strauss HW (2009). One step closer to imaging vulnerable plaque in the coronary arteries. *Journal of Nuclear Medicine*.

[28] Joshi F, Rosenbaum D, Bordes S, Rudd JH (2011). Vascular imaging with positron emission tomography. *Journal of Internal Medicine*.

[29] Klocke FJ, Baird MG, Lorell BH (2003). ACC/AHA/ASNC guidelines for the clinical use of cardiac radionuclide imaging-executive summary: a report of the American College of Cardiology/American Heart Association Task Force on Practice Guidelines (ACC/AHA/ASNC Committee to revise the 1995 guidelines for the clinical use of cardiac radionuclide imaging). *Journal of the American College of Cardiology*.

[30] Udelson JE, Beshansky JR, Ballin DS (2002). Myocardial perfusion imaging for evaluation and triage of patients with suspected acute cardiac ischemia: a randomized controlled trial. *Journal of the American Medical Association*.

[31] Stone GW, Maehara A, Lansky AJ (2011). A prospective natural-history study of coronary atherosclerosis. *The New England Journal of Medicine*.

[32] Von Birgelen C, Klinkhart W, Mintz GS (2001). Plaque distribution and vascular remodeling of ruptured and nonruptured coronary plaques in the same vessel: an intravascular ultrasound study in vivo. *Journal of the American College of Cardiology*.

[33] Kubo T, Maehara A, Mintz GS (2010). The dynamic nature of coronary artery lesion morphology assessed by serial virtual histology intravascular ultrasound tissue characterization. *Journal of the American College of Cardiology*.

[34] Deliargyris EN (2010). Intravascular ultrasound virtual histology derived thin cap fibroatheroma. Now you see it, now you don’t. *Journal of the American College of Cardiology*.

[35] Ino Y, Kubo T, Tanaka A (2011). Difference of culprit lesion morphologies between ST-segment elevation myocardial infarction and non-ST-segment elevation acute coronary syndrome. *Journal of the American College of Cardiology*.

[36] Burke AP, Farb A, Malcom GT, Liang YH, Smialek J, Virmani R (1997). Coronary risk factors and plaque morphology in men with coronary disease who died suddenly. *The New England Journal of Medicine*.

[37] Yonetsu T, Kakuta T, Lee T (2011). In vivo critical fibrous cap thickness for rupture-prone coronary plaques assessed by optical coherence tomography. *European Heart Journal*.

